# BSJPHXF modulates the FGF23-Klotho bone-kidney axis and calcium-phosphorus metabolism in ovariectomized rats with postmenopausal osteoporosis

**DOI:** 10.3389/fendo.2026.1943198

**Published:** 2026-10-23

**Authors:** Huibing Zhang, Yinyin Zhang, Bo Ning, Yanping Lin, Peiyu Wang, Xiaoyu Zhang, Zehua Guo, Genfu Zhu

**Affiliations:** 1 The Third Clinical Medical College, Guangzhou University of Chinese Medicine, Guangzhou, China; 2 The Third Affiliated Hospital of Guangzhou University of Chinese Medicine, Guangzhou, China; 3 The First Clinical Medical College, Henan University of Chinese Medicine, Zhengzhou, China

**Keywords:** BSJPHXF, calcium-phosphorus metabolism, Chinese herbal formula, FGF23-Klotho axis, ovariectomy, postmenopausal osteoporosis

## Abstract

**Objective:**

To investigate the effects of the Kidney-Tonifying, Spleen-Strengthening and Blood-Activating Formula (BSJPHXF, also known as Gukang Formula) on the FGF23-Klotho bone-kidney axis and calcium-phosphorus metabolism in a rat model of postmenopausal osteoporosis.

**Methods:**

Forty SPF female Sprague-Dawley rats were randomly divided into sham-operated, ovariectomized (OVX; model), BSJPHXF and Liuwei Dihuang Wan (LWDHW; positive control) groups (n=10 per group) and treated by gavage for 4 weeks after a 12-week recovery period. Serum calcium and phosphorus were measured by automated biochemical analysis; serum FGF23, parathyroid hormone (PTH) and 1, 25-dihydroxyvitamin D_3_ [1, 25-(OH)_2_D_3_] by ELISA; renal Klotho, NaPi-IIa and 1α-hydroxylase (Cyp27b1) by Western blot; bone microstructure by Micro-CT; and renal histopathology by HE staining. Serum biochemistry and ELISA were performed in nine animals per group, and Western blot, Micro-CT and histology in three animals per group; the animal was the experimental unit.

**Results:**

Compared with sham, OVX rats had lower serum calcium (1.997 ± 0.109 vs. 2.547 ± 0.189 mmol/L) and 1, 25-(OH)_2_D_3_ (102.21 ± 2.87 vs. 201.74 ± 5.85 pg/mL) and higher serum phosphorus (2.019 ± 0.099 vs. 1.355 ± 0.027 mmol/L), FGF23 (366.82 ± 28.36 vs. 159.52 ± 20.39 pg/mL) and PTH (141.63 ± 15.29 vs. 33.91 ± 7.59 pg/mL) (all P<0.001). BSJPHXF attenuated these changes relative to OVX (P<0.001). BSJPHXF and LWDHW did not differ for calcium, FGF23 or PTH, whereas LWDHW produced a greater reduction in serum phosphorus (1.463 ± 0.062 vs. 1.587 ± 0.048 mmol/L, P = 0.0015) and a greater increase in 1, 25-(OH)_2_D_3_ (174.66 ± 7.00 vs. 156.54 ± 13.09 pg/mL, P = 0.0002). On Micro-CT, BSJPHXF, but not LWDHW, improved trabecular bone volume fraction, separation and number versus OVX, and BSJPHXF was superior to LWDHW for all three; trabecular thickness did not differ among groups.

**Conclusion:**

In OVX rats, BSJPHXF attenuated calcium-phosphorus disturbance, with accompanying changes in renal Klotho, NaPi-IIa and Cyp27b1 and in circulating FGF23, PTH and 1, 25-(OH)_2_D_3_, and improved trabecular microarchitecture. As the mechanistically most important endpoints rested on three animals per group and functional renal parameters were not measured, these findings are preliminary and hypothesis-generating.

## 1 Introduction

Osteoporosis (OP) is a systemic metabolic skeletal disorder characterized by reduced bone mass, microarchitectural deterioration and increased fragility, predisposing patients to fragility fractures (1). With an ageing population it has become a major public health burden: more than 200 million people are affected worldwide, and roughly one in three women and one in five men over the age of 50 will sustain an osteoporotic fracture (2, 3). Postmenopausal osteoporosis, caused primarily by the decline in oestrogen after menopause, is the most common form and accounts for much of this burden (4).

The kidney is a principal regulator of mineral homeostasis, and the anti-ageing protein Klotho — expressed predominantly in renal tubular cells — produces a syndrome resembling accelerated human ageing, with vascular calcification, skin atrophy and osteoporosis when deficient (5). Traditional Chinese Medicine (TCM) regards osteoporosis (OP) as rooted in “kidney essence deficiency”, the kidney being believed to govern bones and produce marrow, a framework that has been related to modern concepts of bone-kidney crosstalk including chronic kidney disease and metabolic bone disorder (6). In parallel, molecular research has identified the FGF23-Klotho endocrine pathway as a central regulator of phosphate and vitamin D homeostasis operating across bone and kidney (7, 8).

Klotho suppresses TGF-β, IGF-1, Wnt and NF-κB signalling and thus exerts anti-oxidative, anti-inflammatory, anti-apoptotic and anti-senescence effects (5, 9). FGF23 is a bone-derived hormone secreted mainly by osteoblasts and osteocytes that acts on the kidney via the systemic circulation (10) and binds a receptor complex of FGF receptor 1 (FGFR1) and the co-receptor α-Klotho — the α-Klotho–FGFR1 complex (11) — to regulate 1α-hydroxylase (Cyp27b1) and the sodium-phosphate cotransporters NaPi-IIa/IIc and thereby calcium-phosphorus metabolism (12, 13). Through this bone-kidney loop, circulating FGF23 promotes phosphaturia and lowers 1, 25-(OH)_2_D_3_, protecting the organism from vitamin D excess and coupling renal phosphate handling to bone mineralization and turnover (13, 14).

The endocrine actions of FGF23/α-Klotho on mineral homeostasis should be distinguished from the cell-autonomous roles of FGF ligands in bone ageing and bone marrow mesenchymal stem cell (BMSC) fate. Independent of this axis, FGF2 acts directly on BMSCs to regulate proliferation, senescence and osteogenic differentiation, and disruption of nuclear FGF2 transport and of FGF2-dependent chromatin architecture drives BMSC ageing and impairs bone regeneration (15, 16); reduced α-Klotho, together with declining renal function, has likewise been associated with accelerated bone loss during biological ageing (17). These mechanisms are distinct from the FGF23-α-Klotho endocrine loop examined here.

The Kidney-Tonifying, Spleen-Strengthening and Blood-Activating Formula (BSJPHXF, also named Gukang Formula) is a ten-herb TCM prescription that tonifies kidney and marrow, strengthens the spleen and activates blood circulation; it is used clinically for OP, and its composition and clinical use were evaluated in a systematic review and meta-analysis of Gukang Capsule (18). Pharmacological studies show that BSJPHXF promotes bone formation and inhibits resorption through PI3K/Akt/NF-κB, Wnt/β-catenin and OPG/RANKL/RANK signalling and that it enhances osteogenic activity by inhibiting Beclin-1-induced autophagy (19, 20); the dose used here was derived from the effective range established in our previous work on this formula in PMOP rats (21). LWDHW (Liuwei Dihuang Wan), a classical kidney-tonifying formula, was chosen as the positive control because it is widely used for postmenopausal OP and has been reported to act on the Klotho-FGF23 system (22, 23), making it a clinically relevant TCM comparator. The study was designed to characterize the effects of BSJPHXF, not to establish superiority over LWDHW: where the two did not differ statistically, this reflects the absence of a detected difference in a study not powered for equivalence and does not imply equivalent efficacy.

The effects of BSJPHXF on the FGF23-Klotho axis and its downstream regulation of calcium-phosphorus metabolism remain incompletely characterized. We therefore used the ovariectomized (OVX) rat, a well-established model of oestrogen-deficiency-induced bone loss, to examine these effects and to generate hypotheses for further mechanistic and translational work in postmenopausal osteoporosis.

## 2 Materials and methods

### 2.1 Materials

#### 2.1.1 Animals

Forty SPF female Sprague-Dawley rats weighing 180–220 g on arrival (approximately 8 weeks of age) were obtained from the Laboratory Animal Center of Guangzhou University of Chinese Medicine (production licence No. SCXK (Yue) 2023-0347) and housed in the SPF facility of the same centre (21–25 °C, relative humidity 50–70%, 12-h light/dark cycle, 4–5 per cage, free access to standard chow and water). After one week of acclimatization the experiment began; with a 12-week recovery period and 4 weeks of treatment, the animals were approximately 26 weeks old at the end of the study. The protocol was approved by the Ethics Committee of Guangzhou University of Chinese Medicine (approval No. 20250220006) and complied with the Guidelines for the Welfare and Ethics of Laboratory Animals.

All 40 rats were randomized to the four groups (10 per group) before any intervention. Three rats died and were excluded from all analyses because no endpoint data could be obtained (one OVX rat from postoperative infection; one rat each in the BSJPHXF and LWDHW groups from improper gavage technique); no rat was excluded on the basis of its results. Blood was collected from nine animals per group, and within each group three animals each were assigned to Micro-CT, Western blot and renal histology (12 animals per technique); the three subsets did not overlap, so 3 + 3 + 3 = 9 animals per group, each animal contributing one tissue-based technique in addition to its blood sample. Because only nine animals were available in each surgical group, nine per group were analysed throughout to keep the groups balanced, and the additional sham-operated animal was not used for any endpoint. The serum biochemistry and ELISA data are therefore reported at n=9 per group and the Micro-CT, Western blot and histological data at n=3 per group (see Statistical Analysis); this small sample for the morphological and molecular endpoints is a major limitation, addressed in the Discussion.

#### 2.1.2 Drugs

BSJPHXF comprises ten herbs: Psoralea corylifolia (Buguzhi), Ziziphus jujuba (Dazao), Astragalus membranaceus (Huangqi), Angelica sinensis (Danggui), Cistanche deserticola (Roucongrong), Paeonia lactiflora (Baishao), Epimedium brevicornu (Yinyanghuo), Cuscuta chinensis (Tusizi), Rehmannia glutinosa (Shudihuang) and Salvia miltiorrhiza (Danshen). The relative proportions (total 139 parts) are Buguzhi 13, Yinyanghuo 16, Roucongrong 16, Shudihuang 16, Baishao 13, Huangqi 16, Tusizi 16, Danshen 16, Danggui 11 and Dazao 6. Herbs were purchased from the pharmacy of the Third Affiliated Hospital of Guangzhou University of Chinese Medicine and authenticated by a licensed pharmacist. A single lyophilised batch was prepared according to the hospital’s standardized protocol (batch No. 20250312); the aqueous extract was freeze-dried and the principal quality markers of each herb were determined by HPLC–MS, with an acceptance limit of ±20% of the nominal content. Marker compounds are listed in **Supplementary Table S2** (e.g. paeoniflorin for Baishao; astragaloside IV and calycosin-7-glucoside for Huangqi; icariin for Yinyanghuo; psoralen and isopsoralen for Buguzhi; danshensu/salvianolic acid B and tanshinone IIA for Danshen; ferulic acid for Danggui). LWDHW (Liuwei Dihuang Wan; Beijing Tongrentang Technology Development Co., Ltd., Beijing, China; batch No. 240301) was purchased from a licensed pharmacy.

Doses were converted from clinical to rat doses by body-surface-area scaling (standard human-to-rat factor 6.3, or the commonly simplified 6.25), as described in the classical monographs *Pharmacological Experimental Methodology* and *Research Methods in Chinese Materia Medica Pharmacology*. The adult human dose of BSJPHXF is 28.6 g/d (from our group’s clinical experience and previous published work), giving (28.6/60) × 6.25 = 2.979 g/(kg·d) for a 60-kg adult; the LWDHW dose of 0.1575 g/(kg·d) was adopted from a published ovariectomized-rat study of LWDHW in postmenopausal osteoporosis (24). The rat doses used were therefore 2.979 g/(kg·d) for BSJPHXF and 0.1575 g/(kg·d) for LWDHW.

#### 2.1.3 Major reagents and instruments

Sodium pentobarbital (3%); ampicillin sodium (Macklin, Shanghai, China, A800429-25g); 4% paraformaldehyde (Zitangjin Biotech, Shanghai, China, BL539A). ELISA kits: rat FGF23 (Elabscience, Wuhan, China, E-EL-R3031; double-antibody sandwich; detection range 62.5–4000 pg/mL; sensitivity 37.5 pg/mL; intra- and inter-assay CV <10%); rat 1, 25-(OH)_2_D_3_ (Jianglai Bio, Shanghai, China, JL54661; double-antibody sandwich; range 50–1000 pg/mL; sensitivity 0.039 ng/mL; CV <10%); rat PTH (Jianglai Bio; double-antibody sandwich; range 7.5–240 pg/mL; sensitivity 1.0 pg/mL; CV <10%). Primary antibodies: Klotho (Proteintech, Rosemont, IL, USA, 28100-1-AP), NaPi-IIa (Proteintech, 26783-1-AP), Cyp27b1 (Bioss, Beijing, China, bs-14146R) and GAPDH. BCA protein assay kit (Beyotime, Shanghai, China, P0010S); calcium and phosphorus assay kits (Servicebio, Wuhan, China, G4401ZD, G4404ZD). Micro-CT (NEMO, Guangzhou, China, NMC-200), automated biochemical analyser (Rayto, Shenzhen, China, Chemray240), microplate reader (Thermo Fisher Scientific, Multiskan MK3), electrophoresis apparatus (Beijing Liuyi, DYY-6C).

### 2.2 Methods

#### 2.2.1 Animal grouping, randomization, and model establishment

After one week of acclimatization, rats were ear-punched and randomly assigned 1:1:1:1 to four groups (n=10): sham-operated, model (OVX), BSJPHXF and LWDHW. Randomization and blinding: group allocation used a computer-generated random sequence and was performed before any intervention by an investigator (Z.G.) not involved in outcome assessment, with the allocation scheme concealed and no assignment altered on the basis of subsequent results. The investigators who performed surgery, modelling and gavage (H.Z., B.N.) did not measure outcomes; Micro-CT quantification (Y.Z.), Western blot densitometry and HE reading (X.Z.) were performed under blinded conditions using animal identification numbers, with group codes decoded only after all measurements were completed. Blinding therefore applied to outcome measurement and data analysis but not to modelling and treatment. Sham animals underwent bilateral ovarian exposure with excision of the surrounding fat only; the other three groups underwent bilateral ovariectomy under sterile conditions. All rats received intramuscular penicillin (40, 000 U/day) for three days post-surgery, and vaginal smears were examined daily for three days, the absence of mature epithelial cells indicating successful ovariectomy. After a 12-week recovery period, the BSJPHXF group received BSJPHXF 2.979 g/(kg·d) and the LWDHW group LWDHW 0.1575 g/(kg·d) by gavage, while the sham and model groups received an equal volume of saline; all treatments were given once daily for 4 consecutive weeks. Serum oestradiol was not measured; confirmation of ovariectomy rested on the surgical procedure and daily vaginal cytology, and the absence of hormonal confirmation is acknowledged as a limitation (see Discussion).

#### 2.2.2 Sample collection

After the final administration and a 12-h fast, rats were anaesthetized with 3% sodium pentobarbital (30 mg/kg, i.p.) and blood was collected from the abdominal aorta; animals were then euthanized with an overdose of sodium pentobarbital (150 mg/kg, i.p.), death being confirmed by cessation of spontaneous breathing and absence of a palpable heartbeat. Serum was separated by centrifugation (3, 500 r/min, 10 min, 4 °C) and stored at −80 °C. Femurs and kidneys were rapidly dissected, snap-frozen in liquid nitrogen and stored at −80 °C. Functional renal markers (serum creatinine, blood urea nitrogen or urinary protein) were not measured in this study, and this is acknowledged as a limitation (see Discussion).

#### 2.2.3 Serum calcium and phosphorus measurement

Serum calcium and phosphorus were quantified on an automated biochemical analyser with the following settings: calcium — wavelength 630 nm, sample volume 2.5 μL, reagent volume 300 μL, reaction time 300 s; phosphorus — wavelength 340 nm, sample volume 3 μL, reagent volume 300 μL, reaction time 300 s.

#### 2.2.4 ELISA for FGF23, PTH and 1, 25-(OH)_2_D_3_

Serum FGF23, PTH and 1, 25-(OH)_2_D_3_ were measured in the same serum samples with commercial kits according to the manufacturers’ protocols (assay type and performance characteristics in Section 2.1.3). Briefly, 100 μL of standard or sample was incubated at 37 °C for 90 min; after washing, 100 μL of enzyme conjugate was added for 30 min at 37 °C; after further washing, 90 μL of chromogen A and 50 μL of chromogen B were added and the plate was incubated in the dark at 37 °C for 10 min; the reaction was stopped and absorbance was read at 450 nm.

#### 2.2.5 Western blot analysis

Kidney tissue (20 mg) was minced and lysed in 100 μL of RIPA buffer with protease inhibitors, incubated on ice for 30 min and centrifuged (12, 000 r/min, 10 min, 4 °C). Protein concentration was determined by BCA assay. Samples were boiled with 5× loading buffer and separated by SDS-PAGE (20 μg per lane), transferred to PVDF membranes, blocked with 5% BSA for 60 min and incubated overnight at 4 °C with primary antibodies against Klotho (1:1000), NaPi-IIa (1:1000), Cyp27b1 (1:800) or GAPDH (1:5000). After TBST washing, membranes were incubated with HRP-conjugated secondary antibody (1:20000) for 2 h, and bands were visualized by enhanced chemiluminescence.

The α-Klotho antibody (Proteintech 28100-1-AP) recognises the extracellular domain; membrane-bound full-length Klotho (~130 kDa) and the secretase-cleaved secreted ectodomain (~62–70 kDa) can be resolved, and the single band detected at approximately 62 kDa — the secreted ectodomain, the main circulating and locally acting form — was quantified. The NaPi-IIa antibody (Proteintech 26783-1-AP) detected a ~75 kDa full-length glycosylated protein and a ~30–37 kDa N-terminal fragment; only the ~75 kDa functional brush-border transporter was quantified, and fragments below 37 kDa were treated as degradation products. The molecular weights shown in **Figure 1** (62, 75 and 35 kDa) are consistent with the text, and densitometry was normalized to GAPDH.Uncropped blots for all replicates, with lane order, group assignment and molecular-weight markers,are provided as **Supplementary Figure S1**.

**Figure 1:**
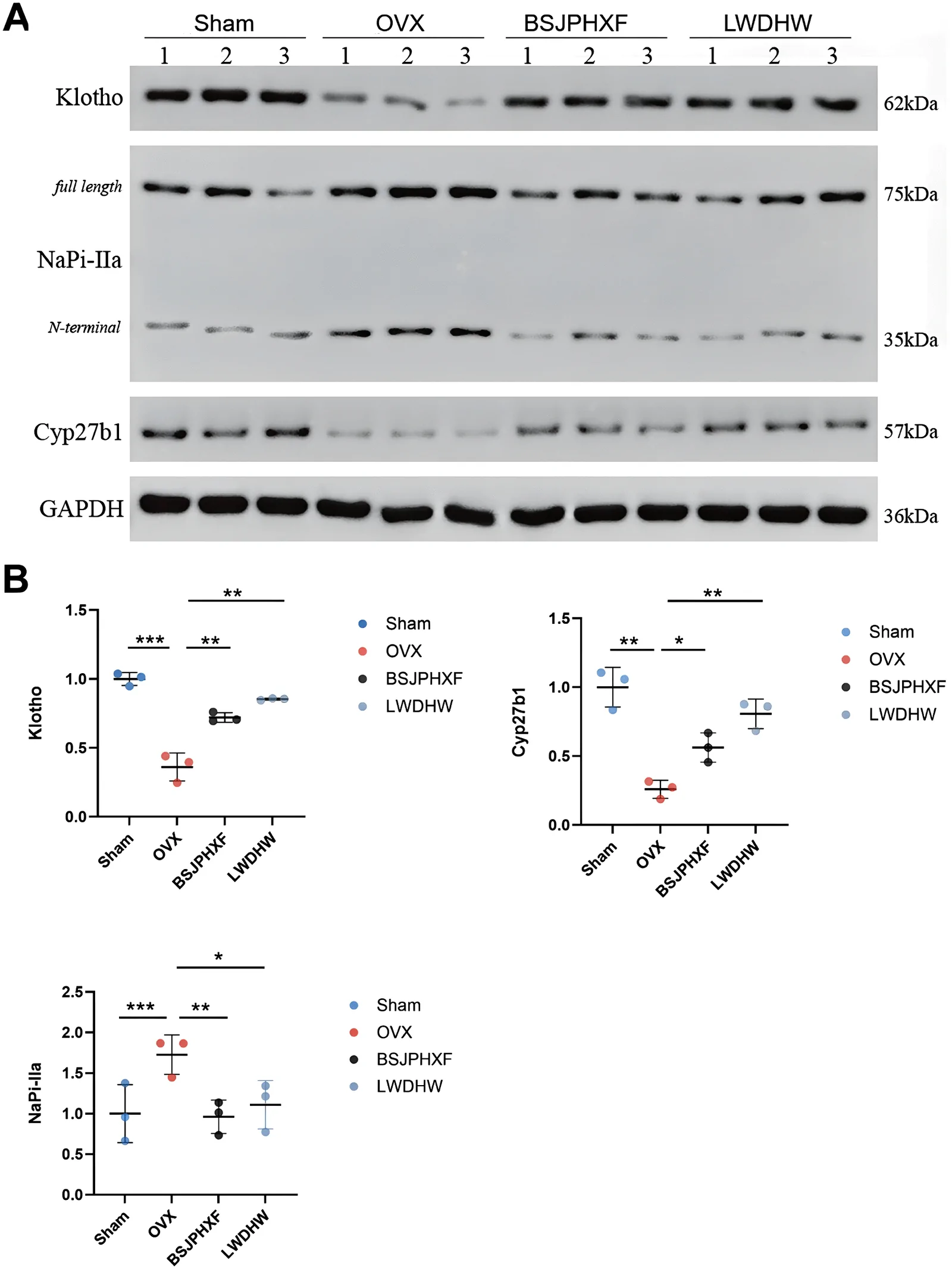
Western blot analysis of renal Klotho, NaPi-IIa, and Cyp27b1 protein expression in each group.**(A)** Representative immunoblots of Klotho, NaPi-IIa, Cyp27b1, and GAPDH (loading control). **(B)** Densitometric quantification of protein expression normalized to GAPDH. Data are mean ± SD (n=3 per group); pairwise comparisons used Tukey’s HSD. Significant differences are indicated by asterisks placed above a bracket that links the two groups being compared (*P<0.05, **P<0.01, ***P<0.001); a bracket is shown only where the corresponding comparison was significant, and the absence of a bracket indicates a non-significant difference. The 62 kDa band corresponds to the secreted ectodomain of α-Klotho and the 75 kDa band to full-length NaPi-IIa; both were quantified as described in Section 2.2.5. The complete, uncropped membranes are shown in **Supplementary Figure S1**.

#### 2.2.6 Micro-CT assessment of bone microstructure

The distal femoral metaphysis was scanned with a Micro-CT system (NEMO, Guangzhou, China; model NMC-200) and reconstructed and analysed with CTvox (Bruker micro-CT, Kontich, Belgium). Acquisition parameters: tube voltage 70 kV, tube current 114 μA, 0.5 mm aluminium filter, integration time 300 ms, 360° rotation, isotropic voxel size 20 μm. Reconstruction and segmentation: Feldkamp back-projection; the trabecular region of interest comprised 100 contiguous slices (2.0 mm) of secondary spongiosa beginning 0.5 mm proximal to the distal femoral growth plate; contours were drawn manually and a global threshold of 220 mg HA/cm^3^ separated mineralised bone from marrow, the identical threshold and region of interest being applied to every specimen. Trabecular number (Tb.N), thickness (Tb.Th), separation (Tb.Sp) and bone volume fraction (Tb.BV/TV) were quantified in the distal femora of three animals per group.

#### 2.2.7 Histopathological examination

Kidney tissue was fixed in 4% paraformaldehyde, dehydrated through a graded ethanol series, cleared in xylene, embedded in paraffin, sectioned at 5 μm and stained with haematoxylin and eosin; images were captured at ×200 magnification with a Leica DMT1 upright microscope with a Nikon DS-U3 imaging system. No blinded semiquantitative scoring (e.g. a tubular injury score) was applied, so the HE findings are reported as qualitative observations only; histology was assessed in n=3 animals per group and is therefore reported descriptively.

#### 2.2.8 Scatter plot visualization

Scatter plots of serum calcium, phosphorus, FGF23, PTH and 1, 25-(OH)_2_D_3_ were generated in GraphPad Prism 10.3. Each symbol represents one measurement (nine animals per group, n=9) and horizontal bars indicate the mean ± standard deviation (SD).

#### 2.2.9 Statistical analysis

Data are expressed as mean ± standard deviation (SD). The experimental unit was the animal; serum calcium and phosphorus and the ELISA measurements (FGF23, PTH and 1, 25-(OH)_2_D_3_) were obtained in nine animals per group (n=9), whereas Western blot, Micro-CT and renal histology were performed in three animals per group (n=3). The rationale for this allocation is given under *Sample size* and its consequences are discussed under Limitations. Normality was assessed with the Shapiro–Wilk test and homogeneity of variance with Levene’s test. Groups were compared by one-way analysis of variance (ANOVA); when the overall ANOVA was significant, pairwise comparisons used Tukey’s honestly significant difference (HSD) test, which controls the family-wise error rate (this replaces the Fisher LSD procedure of the original submission). Where variances were unequal, Welch’s ANOVA with the Games–Howell test was used; this applied to 1, 25-(OH)_2_D_3_ (Levene P = 0.0035). Shapiro–Wilk indicated deviation from normality for serum phosphorus in three of the four groups (P = 0.008–0.049) and for FGF23 in one group (LWDHW, P = 0.043); calcium, 1, 25-(OH)2D3 and PTH satisfied the normality assumption (P = 0.099–0.941), and PTH additionally satisfied homogeneity of variance (Levene P = 0.101). Because group sizes were equal and Levene’s test indicated homogeneous variances for serum phosphorus (P = 0.246) — conditions under which the F test is robust to mild departures from normality — one-way ANOVA with Tukey’s HSD was retained as the primary analysis, with the non-parametric Kruskal–Wallis test performed in parallel as a sensitivity analysis (it confirmed the overall group difference for all five analytes, P<0.001 in each case). For every parameter to which both procedures were applied, including 1, 25-(OH)_2_D_3_, Games–Howell and Tukey’s HSD identified the same set of significant pairwise differences; they differed only in the level of significance assigned to the BSJPHXF versus LWDHW contrast for 1, 25-(OH)_2_D_3_ (Tukey P = 0.0002; Games–Howell P = 0.014), which was significant under both tests. Effect sizes are reported as eta-squared (η^2^) and omega-squared (ω^2^) for the ANOVA and as Cohen’s d for pairwise comparisons. A two-sided P value <0.05 was considered statistically significant. Analyses used SPSS 26.0 (IBM Corp., Armonk, NY, USA) and R 4.4.1 (R Foundation for Statistical Computing, Vienna, Austria); graphs were generated with GraphPad Prism 10.3.

Forty rats were allocated to four groups (n=10 per group); three died and 37 remained. The number of animals per tissue-based technique was fixed in advance in the approved protocol — each technique consumes a distinct specimen (distal femur for Micro-CT, kidney for immunoblotting or histology), with no specimen divided between techniques — for reasons of workload and cost rather than on the basis of the results obtained; the resulting two-tier evidence structure, in which the mechanistically most important endpoints rest on the smaller sample, is acknowledged as a limitation. The group size was determined *a priori*. Using G*Power 3.1.9.4 (F test; one-way ANOVA; four groups; two-sided α=0.05; power=0.80) and assuming a large effect size (f=0.60) consistent with our previous studies of this formula in ovariectomized rats, nine animals per group (36 in total) were required; to allow for possible post-surgical loss, 40 rats (ten per group) were enrolled, three died and 36 (nine per group) completed the analyses reported here. The n=3 subsets used for the morphological and molecular endpoints were not powered for those comparisons, at which sample size the parametric assumptions cannot be reliably evaluated and a single outlier can shift the group mean; those results should therefore be considered preliminary and hypothesis-generating (see Discussion). In addition, the absence of a statistically significant difference between BSJPHXF and LWDHW should not be interpreted as evidence of equivalent efficacy, as the study was not powered for equivalence testing.

## 3 Results

### 3.1 Micro-CT findings

Trabecular microarchitecture of the distal femoral metaphysis differed markedly among groups (**Figure 2**). Relative to sham, the OVX group showed lower trabecular bone volume fraction (Tb.BV/TV 0.087 ± 0.007 vs. 0.292 ± 0.031) and trabecular number (Tb.N 2.595 ± 0.132 vs. 3.613 ± 0.168 mm^-1^) and higher trabecular separation (Tb.Sp 0.302 ± 0.021 vs. 0.187 ± 0.018 mm) (all P<0.001; one-way ANOVA F (3, 8)=27.44, 25.46 and 25.89 respectively, P<0.001), consistent with trabecular bone loss and microarchitectural deterioration after ovariectomy. Trabecular thickness (Tb.Th) did not differ among the four groups (0.091 ± 0.005, 0.084 ± 0.003, 0.087 ± 0.002 and 0.089 ± 0.005 mm for sham, OVX, BSJPHXF and LWDHW; F (3, 8)=1.50, P = 0.288; all pairwise comparisons P>0.2 by Tukey’s HSD).

**Figure 2:**
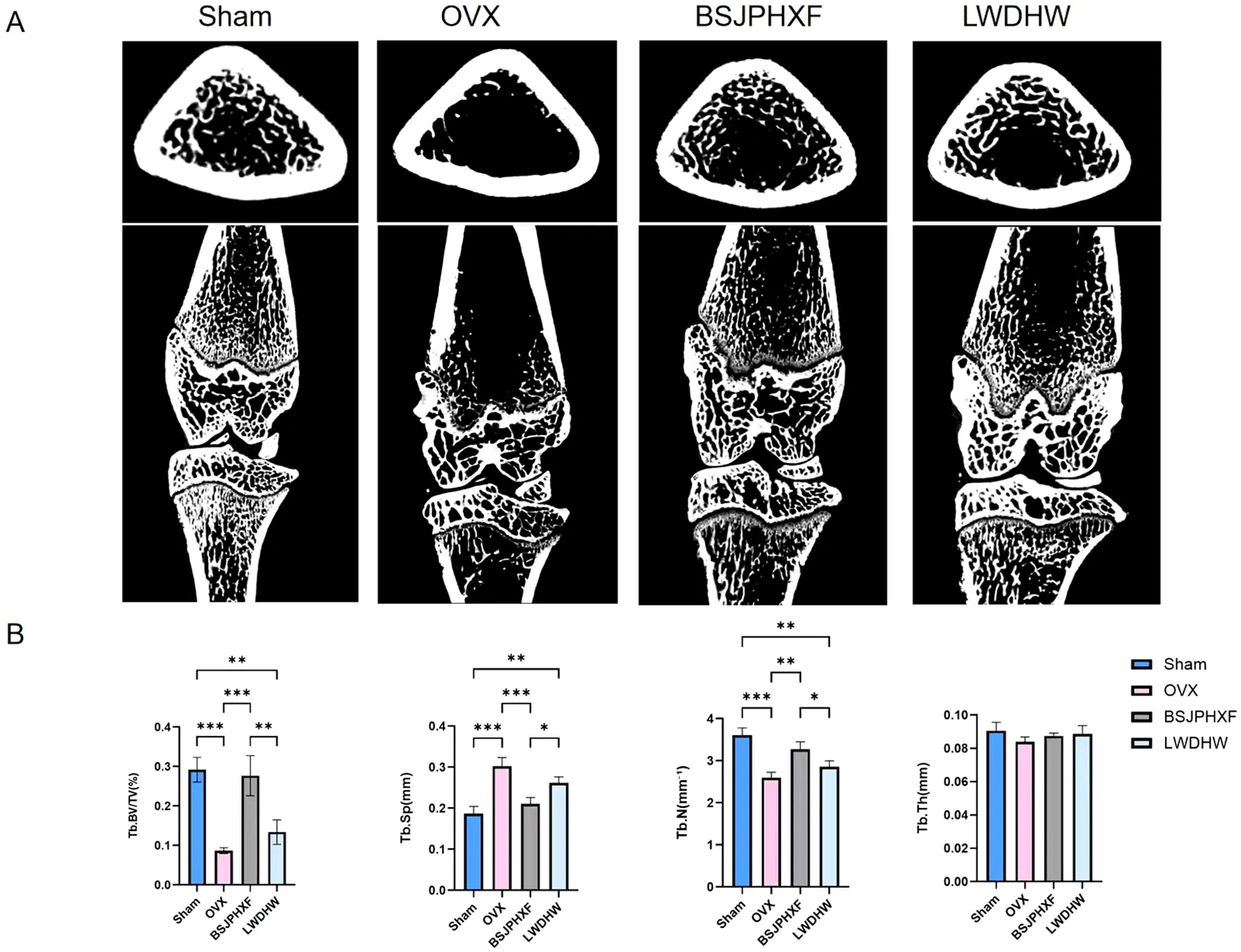
Three-dimensional reconstructed Micro-CT images and trabecular bone microstructure parameters of the distal femur in each group. Representative three-dimensional Micro-CT reconstructions of the distal femoral metaphysis (top row) and quantitative analysis of trabecular bone parameters (bottom row; Tb.BV/TV, Tb.Sp, Tb.N and Tb.Th) in sham-operated, model (OVX: ovariectomized), BSJPHXF and LWDHW groups. Significant differences are indicated by asterisks placed above a horizontal line that links the two groups being compared (*P<0.05, **P<0.01, ***P<0.001, ****P<0.0001); a line is shown only where the corresponding comparison was significant, and the absence of a line indicates a non-significant difference. Tb.Th is shown without markers because no pairwise comparison approached significance (all P>0.2). BSJPHXF differed significantly from OVX and from LWDHW for Tb.BV/TV, Tb.Sp and Tb.N, whereas LWDHW did not differ significantly from OVX for any of these parameters. Tb.BV/TV, trabecular bone volume fraction; Tb.Sp, trabecular separation; Tb.N, trabecular number; Tb.Th, trabecular thickness.

Treatment effects differed between the two formulae. BSJPHXF significantly reversed the OVX-induced changes in all three affected parameters (Tb.BV/TV 0.277 ± 0.051; Tb.Sp 0.211 ± 0.015 mm; Tb.N 3.278 ± 0.174 mm^-1^; all P<0.01 vs. OVX), whereas LWDHW did not significantly reverse any of these parameters relative to OVX (Tb.BV/TV 0.134 ± 0.031, P = 0.382; Tb.Sp 0.261 ± 0.015 mm, P = 0.085; Tb.N 2.861 ± 0.139 mm^-1^, P = 0.228). BSJPHXF was significantly superior to LWDHW for all three parameters (Tb.BV/TV P = 0.004; Tb.Sp P = 0.032; Tb.N P = 0.043). The overall ANOVA effect sizes were large (Tb.BV/TV η^2^ = 0.911, ω^2^ = 0.869; Tb.Sp η^2^ = 0.907, ω^2^ = 0.862; Tb.N η^2^ = 0.905, ω^2^ = 0.859), although at this sample size these estimates are themselves imprecise. These analyses used n=3 per group and Tukey’s HSD throughout (**Table 1**) and should be interpreted as preliminary.

**Table 1:** Micro-CT trabecular bone parameters of the distal femur (n = 3 animals per group).

Parameter	Sham	OVX (model)	BSJPHXF	LWDHW	F (3, 8)	P
Tb.BV/TV	0.292 ± 0.031	0.087 ± 0.007^***^	0.277 ± 0.051^###,††^	0.134 ± 0.031^**^	27.44	<0.001
Tb.Sp (mm)	0.187 ± 0.018	0.302 ± 0.021^***^	0.211 ± 0.015^##,†^	0.261 ± 0.015^**^	25.89	<0.001
Tb.N (mm^-1^)	3.613 ± 0.168	2.595 ± 0.132^***^	3.278 ± 0.174^##,†^	2.861 ± 0.139^**^	25.46	<0.001
Tb.Th (mm)	0.091 ± 0.005	0.084 ± 0.003	0.087 ± 0.002	0.089 ± 0.005	1.50	0.288

Data are mean ± SD (n=3 per group). One-way ANOVA (df=3, 8) with Tukey’s HSD for all pairwise comparisons. *P<0.05, **P<0.01, ***P<0.001 vs. sham; ^#^P<0.05, ^##^P<0.01, ^###^P<0.001 vs. OVX; ^†^P<0.05, ^††^P<0.01, ^†††^P<0.001 BSJPHXF vs. LWDHW (daggers are shown in the BSJPHXF column). Absence of a symbol indicates a non-significant difference; Tb.Th is shown without markers because no pairwise comparison approached significance (all P>0.2). Group means and the exact Tukey P values for the BSJPHXF-versus-OVX, LWDHW-versus-OVX and BSJPHXF-versus-LWDHW comparisons are given in Results 3.1. Tb.BV/TV, trabecular bone volume fraction; Tb.Sp, trabecular separation; Tb.N, trabecular number; Tb.Th, trabecular thickness; OVX, ovariectomized; BSJPHXF, Kidney-Tonifying, Spleen-Strengthening and Blood-Activating Formula; LWDHW, Liuwei Dihuang Wan.

### 3.2 Serum calcium, phosphorus, FGF23, PTH, and 1, 25-(OH)_2_D_3_ levels

Serum calcium, phosphorus, FGF23, PTH and 1, 25-(OH)_2_D_3_ distributions are shown in **Figure 3** and summarized in **Table 2**. OVX rats had lower serum calcium (1.997 ± 0.109 vs. 2.547 ± 0.189 mmol/L) and higher serum phosphorus (2.019 ± 0.099 vs. 1.355 ± 0.027 mmol/L) than sham animals (both P<0.0001), indicating disturbed calcium-phosphorus metabolism. BSJPHXF increased serum calcium (2.288 ± 0.141 mmol/L; P = 0.0006 vs. OVX) and reduced serum phosphorus (1.587 ± 0.048 mmol/L; P<0.0001 vs. OVX); LWDHW likewise increased calcium (2.374 ± 0.105 mmol/L) and reduced phosphorus (1.463 ± 0.062 mmol/L) (both P<0.0001 vs. OVX). Calcium did not differ between the two treatments (P = 0.563), whereas LWDHW reduced serum phosphorus significantly more than BSJPHXF (P = 0.0015). Serum phosphorus showed some deviation from normality, but variances were homogeneous (Levene P = 0.246) and Kruskal–Wallis confirmed the overall group difference (H = 31.67, df=3, P<0.0001); the pairwise comparisons reported are therefore those of the prespecified primary analysis (one-way ANOVA with Tukey’s HSD), and the effect sizes were large (Cohen’s d = 5.56 for BSJPHXF vs. OVX and 2.25 for BSJPHXF vs. LWDHW). As noted above, the absence of a statistically significant difference does not establish equivalence.

**Figure 3:**
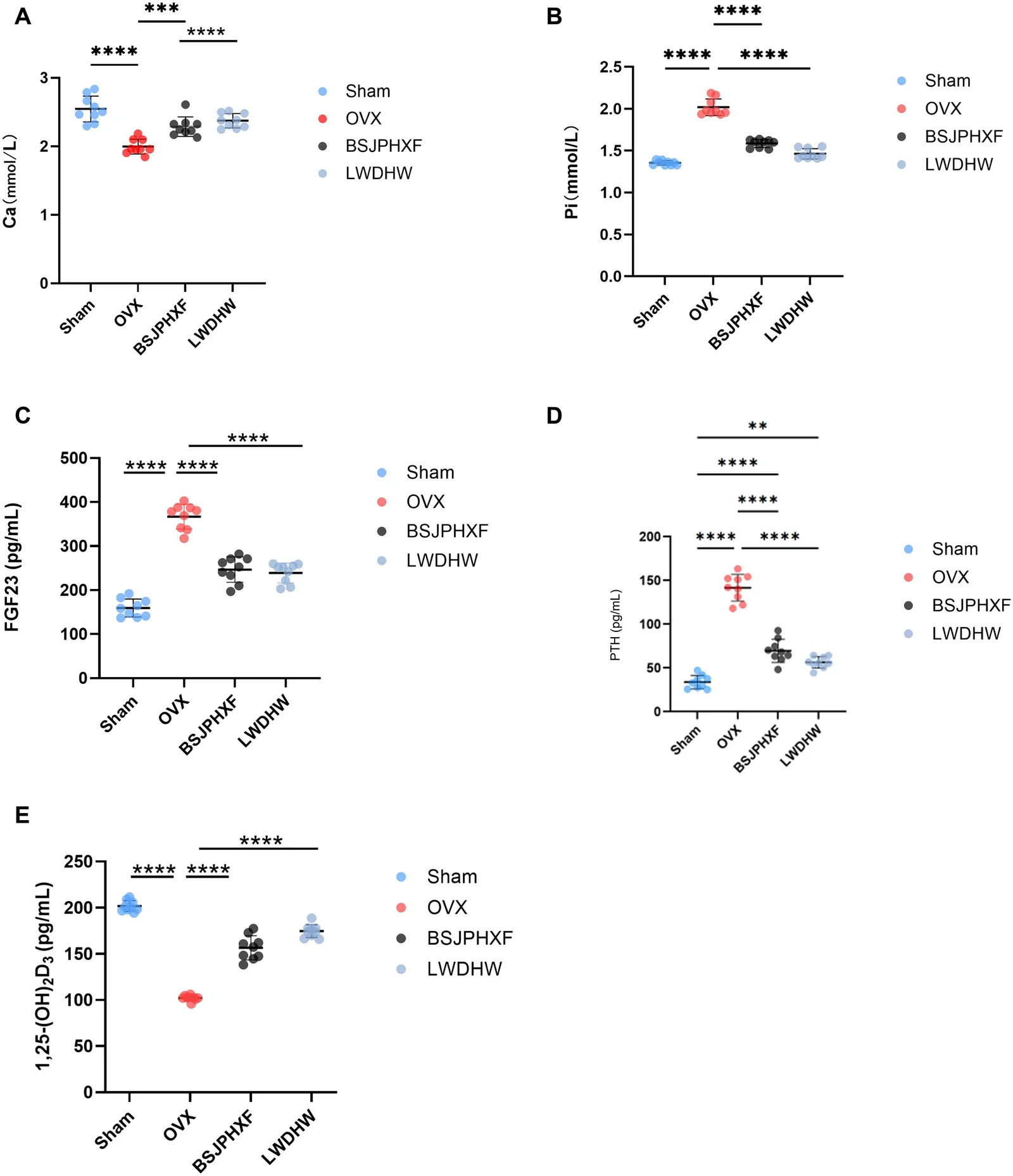
Scatter plots showing serum calcium, phosphorus, FGF23, PTH and 1, 25-(OH)_2_D_3_ levels in each group. Serum calcium, phosphorus, FGF23, PTH and 1, 25-(OH)_2_D_3_ levels in sham-operated, model (OVX: ovariectomized), BSJPHXF, and LWDHW groups. The five panels are arranged in a 2 × 3 grid with the bottom-right position left empty: **(A)** serum calcium; **(B)** serum phosphorus; **(C)** FGF23; **(D)** PTH; **(E)** 1, 25-(OH)_2_D_3_. Each symbol represents one animal (n=9 animals per group), and the horizontal bars indicate the mean ± SD. Observed individual values ranged from 1.85 to 2.84 mmol/L for calcium, 1.33 to 2.19 mmol/L for phosphorus, 137 to 403 pg/mL for FGF23, 25 to 163 pg/mL for PTH and 96 to 212 pg/mL for 1, 25-(OH)_2_D_3_. Data are shown as mean ± SD (n=3 per group); pairwise comparisons used Tukey’s HSD. Significant differences are indicated by asterisks placed above a horizontal line that links the two groups being compared (*P<0.05, **P<0.01, ***P<0.001); a line is shown only where the corresponding comparison was significant, and the absence of a line indicates a non-significant difference. All pairwise comparisons were made with Tukey’s HSD; for 1, 25-(OH)_2_D_3_, Welch’s ANOVA was used as the omnibus test and the Games–Howell test identified the same set of significant pairwise differences.

**Table 2:** Serum biochemical and endocrine parameters in the four groups (n = 9 animals per group).

Parameter	Sham	OVX (model)	BSJPHXF	LWDHW	F (3, 32)	P
Calcium (mmol/L)	2.547 ± 0.189	1.997 ± 0.109^****^	2.288 ± 0.141^**,###^	2.374 ± 0.105^####^	24.28	<0.0001
Phosphorus (mmol/L)	1.355 ± 0.027	2.019 ± 0.099^****^	1.587 ± 0.048^****,####,††^	1.463 ± 0.062^**,####^	183.78	<0.0001
FGF23 (pg/mL)	159.52 ± 20.39	366.82 ± 28.36^****^	246.65 ± 28.96^****,####^	238.88 ± 22.47^****,####^	102.65	<0.0001
PTH (pg/mL)	33.91 ± 7.59	141.63 ± 15.29^****^	69.60 ± 13.20^****,####^	56.39 ± 6.52^**,####^	153.56	<0.0001
1, 25-(OH)_2_D_3_ (pg/mL)	201.74 ± 5.85	102.21 ± 2.87^****^	156.54 ± 13.09^****,####,†††^	174.66 ± 7.00^****,####^	798.51^‡^	<0.0001

Data are mean ± SD (n=9 per group). One-way ANOVA (df=3, 32) with Tukey’s HSD for pairwise comparisons. *P<0.05, **P<0.01, ***P<0.001, ****P<0.0001 vs. sham; ^#^P<0.05, ^##^P<0.01, ^###^P<0.001, ^####^P<0.0001 vs. OVX; ^†^P<0.05, ^††^P<0.01, ^†††^P<0.001 BSJPHXF vs. LWDHW (daggers are shown in the BSJPHXF column). Absence of a symbol indicates a non-significant difference (e.g. calcium, LWDHW vs. sham, P = 0.061). ‡For 1, 25-(OH)_2_D_3_ the Levene test indicated unequal variances (P = 0.0035), so Welch’s F (df=3, 15.9) is given; Games–Howell identified the same significant comparisons as Tukey’s HSD. Exact P values, effect sizes and the Shapiro–Wilk, Levene, Welch and Kruskal–Wallis results are in **Supplementary Table S1**. OVX, ovariectomized; BSJPHXF, Kidney-Tonifying, Spleen-Strengthening and Blood-Activating Formula; LWDHW, Liuwei Dihuang Wan; PTH, parathyroid hormone.

OVX rats also had higher FGF23 (366.82 ± 28.36 vs. 159.52 ± 20.39 pg/mL) and PTH (141.63 ± 15.29 vs. 33.91 ± 7.59 pg/mL) and lower 1, 25-(OH)_2_D_3_ (102.21 ± 2.87 vs. 201.74 ± 5.85 pg/mL) than sham animals (all P<0.0001), consistent with altered bone-kidney endocrine signalling and with secondary hyperparathyroidism. BSJPHXF and LWDHW both reduced FGF23 (246.65 ± 28.96 and 238.88 ± 22.47 pg/mL) and PTH (69.60 ± 13.20 and 56.39 ± 6.52 pg/mL) and increased 1, 25-(OH)_2_D_3_ (156.54 ± 13.09 and 174.66 ± 7.00 pg/mL) relative to OVX (all P<0.0001). The two treatments did not differ for FGF23 (P = 0.915) or PTH (P = 0.082), whereas LWDHW increased 1, 25-(OH)_2_D_3_ significantly more than BSJPHXF (P = 0.0002). Because Levene’s test indicated unequal variances for 1, 25-(OH)_2_D_3_ (P = 0.0035), Welch’s ANOVA (F = 798.51, df = 3, 15.9, P<0.0001) with the Games–Howell test was used for this parameter; the Games–Howell test identified the same set of significant pairwise differences as Tukey’s HSD, differing only in the level assigned to the BSJPHXF versus LWDHW contrast (Tukey P = 0.0002; Games–Howell P = 0.014). The overall ANOVA effect sizes were large (serum calcium η^2^ = 0.695, ω^2^ = 0.660; phosphorus η^2^ = 0.945, ω^2^ = 0.938; FGF23 η^2^ = 0.906, ω^2^ = 0.894; 1, 25-(OH)_2_D_3_ η^2^ = 0.958, ω^2^ = 0.953; PTH η^2^ = 0.935, ω^2^ = 0.927).

### 3.3 Renal protein expression of Klotho, NaPi-IIa, and Cyp27b1

Renal Klotho and Cyp27b1 band intensities were lower and NaPi-IIa intensity higher in the OVX group than in sham animals (**Figure 1A**), and densitometric quantification (**Figure 1B**) confirmed downregulation of Klotho and Cyp27b1 and upregulation of NaPi-IIa (all P<0.01); treatment with BSJPHXF or LWDHW produced the reverse pattern (all P<0.01 vs. OVX). Band identities and molecular weights are specified in Section 2.2.5 and the **Figure 1** legend. These analyses used n=3 animals per group and the results should therefore be regarded as preliminary.

### 3.4 Renal histopathological observations

Renal histology (**Figure 4**) showed intact glomeruli and regularly arranged tubules with normal epithelial cell morphology in sham animals, whereas the OVX group showed vacuolar degeneration of tubular epithelial cells, dilation of some tubules and occasional proteinaceous casts within the lumina; BSJPHXF and LWDHW were associated with milder tubular alterations, improved tubular arrangement and reduced vacuolation. These observations are descriptive, were obtained from a single representative section per animal in n=3 animals per group, and were not scored semiquantitatively or accompanied by functional renal measurements; they are therefore interpreted only as qualitative histopathological observations (see Discussion).

**Figure 4:**
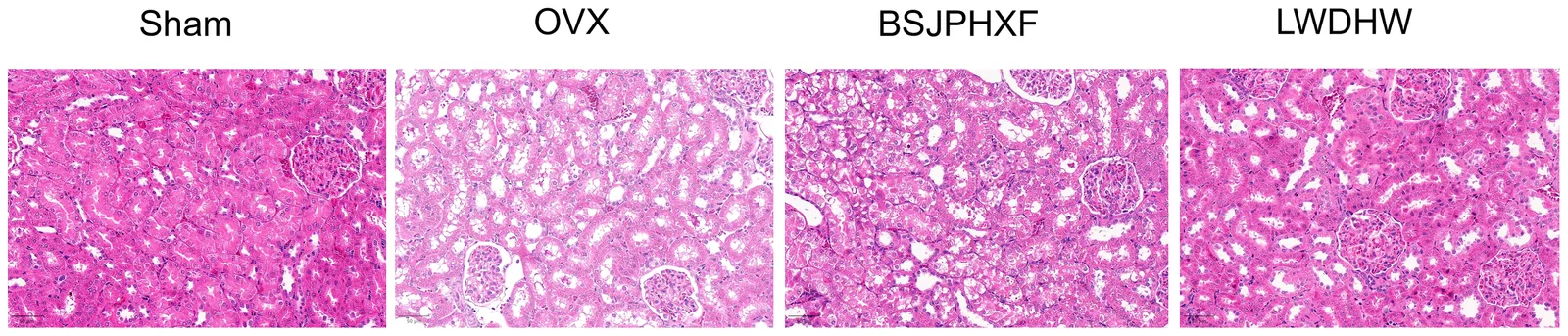
Representative HE-stained kidney sections from each group (magnification ×200). Sham group: intact glomeruli and regularly arranged tubules with normal epithelial cells. Model (OVX: ovariectomized) group: vacuolar degeneration of tubular epithelial cells, tubular dilation, and occasional proteinaceous casts. BSJPHXF and LWDHW groups: milder tubular alterations with improved tubular arrangement. Scale bar = 50 μm. These images are representative of n=3 animals per group and are presented as qualitative observations.

## 4 Discussion

In the present study, ovariectomized rats showed elevated serum FGF23, reduced 1, 25-(OH)_2_D_3_ and elevated PTH with disturbed calcium–phosphorus homeostasis, together with reduced renal Klotho and Cyp27b1 and increased NaPi-IIa; BSJPHXF partially reversed these changes and improved trabecular microarchitecture. Postmenopausal osteoporosis is the most common form of osteoporosis, and disturbed calcium–phosphorus metabolism contributes importantly to postmenopausal bone loss. Calcium and phosphorus are stored in bone mainly as hydroxyapatite and calcium phosphate, and their equilibrium is maintained largely by the kidney through the FGF23-α-Klotho endocrine axis, which coordinates mineral handling across the kidney, intestine and bone (9). FGF23 is a bone-derived hormone that acts on renal tubular cells through the α-Klotho–FGFR1 complex to promote phosphaturia and reduce circulating 1, 25-(OH)_2_D_3_ (7, 8, 11, 13). Klotho is both the essential co-receptor for FGF23 and a soluble hormone-like factor that modulates Wnt, IGF-1, NF-κB and TGF-β signalling, thereby influencing oxidative stress, inflammation and tissue senescence (5, 9, 25); deficiency of Klotho or FGF23 causes hyperphosphataemia, vascular calcification and premature ageing, whereas Klotho overexpression extends lifespan (5, 9). Mechanistically, the axis lowers circulating phosphate by downregulating renal NaPi-IIa/IIc (12), suppresses Cyp27b1 while inducing Cyp24a1 to reduce 1, 25-(OH)_2_D_3_ synthesis (26), modulates PTH secretion, and influences osteoblast differentiation and bone mineralization, with the calcium-sensing receptor participating through crosstalk with the axis (27).

This endocrine axis should nevertheless be distinguished from the cell-autonomous actions of locally acting FGF ligands in bone. Whereas FGF23 signals through the α-Klotho–FGFR1 complex on renal tubular cells, FGF2 and related ligands act directly on bone marrow stromal and osteogenic cells, regulating proliferation, senescence and lineage commitment through mechanisms that include nuclear transport and chromatin-level control of ribosomal DNA architecture (15, 16); reduced α-Klotho and declining renal function have similarly been linked to accelerated bone loss during biological ageing (17). The present study addresses only the endocrine arm and provides no evidence on these cell-autonomous pathways, and the two should not be conflated.

Traditional Chinese medicine classifies osteoporosis as “bone atrophy” or “bone impediment” and attributes it to kidney essence deficiency, often accompanied by spleen deficiency and blood stasis. The OVX rat, however, models the acute consequences of oestrogen withdrawal and is best regarded as a model of postmenopausal osteoporosis rather than of age-related “kidney essence deficiency” as understood in TCM, and it is not a model of renal osteodystrophy or chronic kidney disease (6, 28), in which the bone phenotype is driven by disturbed mineral metabolism rather than by oestrogen loss. Because oestrogen deficiency accelerates bone remodelling and shifts the balance towards resorption, this is the principal mechanism of bone loss in OVX animals (29). The renal changes in Klotho, NaPi-IIa and Cyp27b1 and the circulating changes in FGF23 and 1, 25-(OH)_2_D_3_ observed here may therefore reflect downstream consequences of altered bone turnover and elevated bone-derived FGF23 rather than a primary renal disorder, and our data cannot distinguish these possibilities.

BSJPHXF (Gukang Formula) derives from modified Yougui Yin and Roucongrong Tang and comprises ten herbs — Psoralea corylifolia, Ziziphus jujuba, Astragalus membranaceus, Angelica sinensis, Cistanche deserticola, Paeonia lactiflora, Epimedium brevicornu, Cuscuta chinensis, Rehmannia glutinosa and Salvia miltiorrhiza — arranged in a sovereign–minister–assistant–messenger hierarchy in which Psoralea corylifolia serves as the sovereign herb. A systematic review and meta-analysis of Gukang Capsule reported improved bone mineral density, faster healing of osteoporotic fractures, reduced bone turnover markers and a higher total effective rate without increased adverse events (18), and experimental work shows that BSJPHXF promotes bone formation through Wnt signalling (20) and osteogenic activity by inhibiting Beclin-1-induced autophagy (19). Several constituent compounds act on related pathways: psoralen through Wnt/β-catenin, BMP2 and oestrogen receptor α activation (20); icariin through BMP signalling and miR-23a-3p-mediated BMP-2/Smad5/Runx2 signalling (30); and Astragalus membranaceus by regulating the vitamin D–FGF23–Klotho axis, increasing Klotho/VDR/CYP27B1 and decreasing FGF23/CYP24A1 in osteoporotic mice (31). The FGF23/Klotho axis also mediates vascular calcification through Wnt7b/β-catenin (32), and other TCM preparations modulate the same axis — modified LWDHW has been reported to improve renal function and provide bone protection through Klotho–FGF23 regulation (22, 23) — while 2, 3, 5, 4′-tetrahydroxystilbene-2-O-β-D-glucoside (TSG) promotes Klotho expression and enhances bone strength (33).

These findings are consistent with the concept that increased FGF23 signalling accompanies reduced vitamin D activation and altered renal phosphate handling (7, 13) and with a state of relative α-Klotho deficiency. The serum PTH data provide indirect support. Physiologically FGF23 suppresses PTH secretion, but this suppression is attenuated when renal and parathyroid α-Klotho availability falls; at the same time, three signals in this model favour PTH secretion — hypocalcaemia (1.997 ± 0.109 vs. 2.547 ± 0.189 mmol/L), hyperphosphataemia (2.019 ± 0.099 vs. 1.355 ± 0.027 mmol/L) and the fall in 1, 25-(OH)_2_D_3_ (102.21 ± 2.87 vs. 201.74 ± 5.85 pg/mL), which releases VDR-mediated inhibition of PTH gene transcription. The rise in PTH in OVX rats (141.63 ± 15.29 vs. 33.91 ± 7.59 pg/mL) is therefore consistent with secondary hyperparathyroidism arising from FGF23 resistance, and its normalization after treatment (BSJPHXF 69.60 ± 13.20 pg/mL; LWDHW 56.39 ± 6.52 pg/mL) closes the loop. Consistent with this, the ovariectomy-induced rise in FGF23 was accompanied by hyperphosphataemia and increased renal NaPi-IIa rather than by the phosphaturia expected from an intact FGF23 effect. Because urinary phosphate excretion, renal Cyp24a1 and downstream FGFR signalling were not measured, FGF23 resistance cannot be formally demonstrated and the direction of causality remains to be established; in a small sample these observations should be regarded as associative rather than causal.

BSJPHXF partially normalized these indices, in the same direction as LWDHW. Because the study was not powered for equivalence testing, the absence of significant differences between the formulae does not establish equivalent efficacy. Where they differed, the differences favoured LWDHW for the circulating mineral parameters — a greater reduction in serum phosphorus (P = 0.0015) and a greater increase in 1, 25-(OH)_2_D_3_ (P = 0.0002) — and BSJPHXF for trabecular microarchitecture (P = 0.004, 0.032 and 0.043 for Tb.BV/TV, Tb.Sp and Tb.N), with no significant difference for serum calcium, FGF23 or PTH. This dissociation suggests that the two formulae are not interchangeable in this model and that the microarchitectural effect of BSJPHXF may not be fully explained by its systemic action on calcium–phosphorus metabolism. Two caveats apply: the assumption of normality was not satisfied for serum phosphorus in three of the four groups and for FGF23 in one group, and with nine animals per group the study has limited power for between-treatment comparisons, so these two differences are reported as suggestive rather than definitive, although the corresponding effect sizes were large (Cohen’s d = 2.25 and −1.73 in favour of LWDHW).

On Micro-CT, BSJPHXF significantly improved trabecular bone volume fraction, separation and number relative to OVX, whereas LWDHW did not reach significance for any of these parameters and trabecular thickness did not differ among groups. For all three affected parameters BSJPHXF was indistinguishable from sham, whereas LWDHW remained significantly below sham (P = 0.0020, 0.0036 and 0.0015). This pattern is consistent with the restored 1, 25-(OH)_2_D_3_ and improved mineral balance required for bone mineralization (13), and again indicates that the formulae are not interchangeable with respect to bone microarchitecture. Because bone histomorphometry, bone turnover markers and mechanical testing were not performed, we cannot conclude that BSJPHXF promotes bone formation or inhibits bone resorption; the absence of a significant LWDHW effect should likewise be read cautiously, as the study was not powered to detect small differences and the LWDHW dose was scaled from a clinical dose. In the kidney, OVX animals showed qualitative tubular alterations on HE staining that were milder after treatment; as these were not scored semiquantitatively in a blinded manner and were not accompanied by functional renal markers, they are reported as descriptive observations only and no conclusion regarding renal protection is drawn.

Integrating these results with previous pharmacological studies (30), BSJPHXF may act through at least two complementary mechanisms: modulation of renal α-Klotho–FGF23 signalling that affects calcium–phosphorus metabolism, and direct effects on bone cells through the Wnt/β-catenin and BMP pathways. This dual action addresses both systemic mineral imbalance and local bone remodelling and may help explain the favourable clinical results reported previously (18). Targeting the FGF23-α-Klotho axis with a multi-component formula is a plausible adjunctive approach for postmenopausal osteoporosis, particularly in patients with coexisting renal impairment or vitamin D insufficiency, although this remains a hypothesis requiring dedicated studies.

The most important limitation is the two-tier evidence structure of the study: the morphological and molecular endpoints that are mechanistically central to our conclusions — Western blot, Micro-CT and renal histology — rest on only three animals per group, whereas the serum biochemistry and ELISA data rest on nine. At n=3 the parametric assumptions cannot be reliably evaluated, statistical power is limited and a single outlier can markedly shift the group mean, so these findings must be regarded as preliminary and hypothesis-generating, and the subsets were not powered for those comparisons. We did not measure renal Cyp24a1 expression or urinary phosphate excretion, nor renal or parathyroid α-Klotho protein or downstream FGFR signalling, so FGF23 resistance could not be demonstrated directly; functional renal parameters and blinded semiquantitative histological scoring were likewise not obtained, so renal structure cannot be linked to renal function, and the absence of bone histopathology, turnover markers and mechanical testing limits any conclusion on bone formation and resorption. Serum sex hormone concentrations were not measured, so the oestrogen-deficient state rests on vaginal cytology and the surgical procedure rather than on hormonal confirmation, and other models of reproductive ageing were not used. In particular, 4-vinylcyclohexene diepoxide (VCD)-induced ovarian failure destroys primordial and primary follicles gradually and therefore reproduces the perimenopausal transition more closely than ovariectomy, while avoiding the surgical trauma and the abrupt withdrawal of ovarian hormones; its disadvantages are a longer induction period, residual chemical ovarian toxicity and much less validation for bone endpoints, which is why the well-characterised OVX model was retained for this first evaluation of BSJPHXF (34). Finally, four groups were compared across multiple endpoints; although Tukey’s HSD controls the family-wise error rate within each endpoint, the analyses remain exploratory and were not adjusted for multiplicity across endpoints, and the absence of pathway-specific inhibitor experiments prevents confirmation of the direct molecular target(s) of BSJPHXF. Adequately powered studies with functional renal and bone endpoints, hormonal confirmation and pathway-specific interventions will be needed to test the mechanisms proposed here.

## 5 Conclusion

In OVX rats, BSJPHXF attenuated calcium-phosphorus metabolic disturbance and was accompanied by changes in serum FGF23, PTH and 1, 25-(OH)_2_D_3_ and in renal Klotho, Cyp27b1 and NaPi-IIa expression, consistent with modulation of the FGF23-α-Klotho bone-kidney endocrine axis; it also improved trabecular bone microarchitecture to a greater extent than LWDHW. Given that the morphological and molecular endpoints were assessed in only three animals per group — although the biochemical endpoints rested on nine animals per group — and given the absence of functional renal and bone endpoints, these findings are preliminary and hypothesis-generating, and further studies with adequate sample sizes are required to establish causality and clinical relevance in postmenopausal osteoporosis.

## Data Availability

The raw data supporting the conclusions of this article will be made available by the authors, without undue reservation. The source images for the Western blots are provided as **Supplementary Figure S1**.

## Supplementary material

Supplementary Figure 1 Uncropped, full-length Western blot images for Cyp27b1, Klotho, NaPi-IIa, and GAPDH. Lanes are shown in group order (sham, OVX, BSJPHXF, LWDHW) with the molecular-weight markers indicated; no lane was duplicated and no image was cropped or spliced.

Supplementary Table 1 Full statistical summary for the serum biochemistry and ELISA parameters, including group means and standard deviations, tests of normality and homogeneity of variance, omnibus statistics, effect sizes and all pairwise comparisons (Tukey’s HSD with Cohen’s d), together with the Welch and Kruskal–Wallis sensitivity analyses.

Supplementary Table 2 Principal quality marker compounds of the ten constituent herbs of BSJPHXF.
