# Supplementary material for: BSJPHXF modulates the FGF23-Klotho bone-kidney axis and calcium-phosphorus metabolism in ovariectomized rats with postmenopausal osteoporosis
Source: Front Endocrinol (Lausanne). 2026 Oct 23;17:1943198. doi: 10.3389/fendo.2026.1943198 (PMC13739526; doi:10.3389/fendo.2026.1943198)
Supplement: Supplementary file 1 [file SupplementaryFile1.docx]

Cyp27b1

Klotho

GAPDH

NaPi-IIa
