# Supplementary material for: BSJPHXF modulates the FGF23-Klotho bone-kidney axis and calcium-phosphorus metabolism in ovariectomized rats with postmenopausal osteoporosis
Source: Front Endocrinol (Lausanne). 2026 Oct 23;17:1943198. doi: 10.3389/fendo.2026.1943198 (PMC13739526; doi:10.3389/fendo.2026.1943198)
Supplement: Supplementary file 2 [file Table1.docx]

Supplementary Table S1. Complete statistical summary for the serum biochemical and endocrine parameters (n = 9 animals per group).

**Descriptive statistics and omnibus tests.**

| Parameter | Sham | OVX (model) | BSJPHXF | LWDHW | F | P |
| --- | --- | --- | --- | --- | --- | --- |
| Serum calcium (mmol/L) | 2.547 ± 0.189 | 1.997 ± 0.109 | 2.288 ± 0.141 | 2.374 ± 0.105 | 24.28 | <0.0001 |
| Serum phosphorus (mmol/L) | 1.355 ± 0.027 | 2.019 ± 0.099 | 1.587 ± 0.048 | 1.463 ± 0.062 | 183.78 | <0.0001 |
| FGF23 (pg/mL) | 159.52 ± 20.39 | 366.82 ± 28.36 | 246.65 ± 28.96 | 238.88 ± 22.47 | 102.65 | <0.0001 |
| PTH (pg/mL) | 33.91 ± 7.59 | 141.63 ± 15.29 | 69.60 ± 13.20 | 56.39 ± 6.52 | 153.56 | <0.0001 |
| 1,25-(OH)_2_D_3_ (pg/mL) | 201.74 ± 5.85 | 102.21 ± 2.87 | 156.54 ± 13.09 | 174.66 ± 7.00 | 798.51 (Welch) | <0.0001 |

F and P are from a one-way ANOVA with 3 and 32 degrees of freedom. For 1,25-(OH)_2_D_3_, Levene’s test indicated unequal variances (P=0.0035) and Welch’s ANOVA was therefore used as the omnibus test (F=798.51, P<0.0001, df1=3, df2=15.92); the ordinary one-way ANOVA gave F=242.14 (P<0.0001). Values are group mean ± SD.

**Pairwise comparisons (Tukey’s HSD P value, with Cohen’s d in parentheses).**

| Contrast | Serum calcium (mmol/L) | Serum phosphorus (mmol/L) | FGF23 (pg/mL) | PTH (pg/mL) | 1,25-(OH)_2_D_3_ (pg/mL) |
| --- | --- | --- | --- | --- | --- |
| Sham vs OVX (model) | <0.0001 (3.58) | <0.0001 (-9.15) | <0.0001 (-8.39) | <0.0001 (-8.92) | <0.0001 (21.59) |
| Sham vs BSJPHXF | 0.0023 (1.56) | <0.0001 (-5.98) | <0.0001 (-3.48) | <0.0001 (-3.31) | <0.0001 (4.46) |
| Sham vs LWDHW | 0.061 (1.13) | 0.0063 (-2.26) | <0.0001 (-3.70) | 0.0010 (-3.18) | <0.0001 (4.20) |
| OVX (model) vs BSJPHXF | 0.0006 (-2.31) | <0.0001 (5.56) | <0.0001 (4.19) | <0.0001 (5.04) | <0.0001 (-5.73) |
| OVX (model) vs LWDHW | <0.0001 (-3.53) | <0.0001 (6.74) | <0.0001 (5.00) | <0.0001 (7.25) | <0.0001 (-13.54) |
| BSJPHXF vs LWDHW | 0.563 (-0.69) | 0.0015 (2.25) | 0.915 (0.30) | 0.082 (1.27) | 0.0002 (-1.73) |

All pairwise comparisons were made with Tukey’s HSD. Cohen’s d was calculated with the pooled SD. PTH values for the two treatment groups did not differ (P=0.082), and FGF23 did not differ between BSJPHXF and LWDHW (P=0.915); by contrast, serum phosphorus and 1,25-(OH)_2_D_3_ were significantly better preserved by LWDHW than by BSJPHXF (P=0.0015 and P=0.0002, respectively).

**Assumptions, effect sizes and sensitivity analyses.**

| Parameter | Shapiro–Wilk P (per group) | Levene P | Kruskal–Wallis H (P) | Welch F (P) | η_2_ | ω_2_ |
| --- | --- | --- | --- | --- | --- | --- |
| Serum calcium (mmol/L) | 0.6763 / 0.3419 / 0.1026 / 0.2376 | 0.3955 | 24.59 (<0.0001) | 26.12 (<0.0001) | 0.695 | 0.660 |
| Serum phosphorus (mmol/L) | 0.1075 / 0.0184 / 0.0489 / 0.0082 | 0.2460 | 31.67 (<0.0001) | 148.55 (<0.0001) | 0.945 | 0.938 |
| FGF23 (pg/mL) | 0.4003 / 0.3571 / 0.5171 / 0.0426 | 0.8351 | 29.73 (<0.0001) | 98.97 (<0.0001) | 0.906 | 0.894 |
| PTH (pg/mL) | 0.5865 / 0.6962 / 0.9412 / 0.5633 | 0.1007 | 30.88 (<0.0001) | 113.50 (<0.0001) | 0.935 | 0.927 |
| 1,25-(OH)_2_D_3_ (pg/mL) | 0.4621 / 0.0988 / 0.7578 / 0.4396 | 0.0035 | 31.67 (<0.0001) | 798.51 (<0.0001) | 0.958 | 0.953 |

Normality was assessed with the Shapiro–Wilk test and homogeneity of variance with Levene’s test (median-centred); the four Shapiro–Wilk P values are given in group order (Sham, OVX, BSJPHXF, LWDHW). The Kruskal–Wallis test and Welch’s ANOVA were run as sensitivity analyses: the Kruskal–Wallis test confirmed the overall group difference for all five analytes (P<0.001 in each case), and the Games–Howell post hoc test identified the same set of significant pairwise differences as Tukey’s HSD (the two tests differed only in the level of significance assigned to the BSJPHXF versus LWDHW contrast for 1,25-(OH)_2_D_3_: Tukey P=0.0002, Games–Howell P=0.014, significant under both). The rank-based (Dunn) post hoc test is markedly less sensitive than Tukey’s HSD at this sample size and with these variance patterns and was therefore not used for pairwise inference. η_2_ and ω_2_ were calculated from the sums of squares of the one-way ANOVA. All five analytes satisfied the normality assumption except serum phosphorus, for which Shapiro–Wilk gave P<0.05 in three of the four groups; PTH satisfied both assumptions (Shapiro–Wilk P=0.563–0.941; Levene P=0.101), so its parametric analysis is fully valid and its raw measurements are given in Supplementary Data S1.
