# Supplementary material for: BSJPHXF modulates the FGF23-Klotho bone-kidney axis and calcium-phosphorus metabolism in ovariectomized rats with postmenopausal osteoporosis
Source: Front Endocrinol (Lausanne). 2026 Oct 23;17:1943198. doi: 10.3389/fendo.2026.1943198 (PMC13739526; doi:10.3389/fendo.2026.1943198)
Supplement: Supplementary file 3 [file Table2.docx]

Supplementary Table S2. Principal quality marker compounds of the ten constituent herbs of BSJPHXF.

| No. | Herb (Latin name) | Chinese name | Proportion (parts) | Principal quality marker compound(s) | Method |
| --- | --- | --- | --- | --- | --- |
| 1 | Psoralea corylifolia L. | 补骨脂 (Buguzhi) | 13 | Psoralen; isopsoralen (angelicin); bakuchiol | HPLC–MS |
| 2 | Ziziphus jujuba Mill. | 大枣 (Dazao) | 6 | Jujuboside A; jujuboside B; betulinic acid | HPLC–MS |
| 3 | Astragalus membranaceus (Fisch.) Bge. | 黄芪 (Huangqi) | 16 | Astragaloside IV; calycosin-7-O-β-D-glucoside | HPLC–MS |
| 4 | Angelica sinensis (Oliv.) Diels | 当归 (Danggui) | 11 | Ferulic acid; ligustilide | HPLC–MS |
| 5 | Cistanche deserticola Y. C. Ma | 肉苁蓉 (Roucongrong) | 16 | Echinacoside; acteoside (verbascoside) | HPLC–MS |
| 6 | Paeonia lactiflora Pall. | 白芍 (Baishao) | 13 | Paeoniflorin | HPLC–MS |
| 7 | Epimedium brevicornu Maxim. | 淫羊藿 (Yinyanghuo) | 16 | Icariin; epimedin C | HPLC–MS |
| 8 | Cuscuta chinensis Lam. | 兔丝子 (Tusizi) | 16 | Hyperoside; rutin; quercetin | HPLC–MS |
| 9 | Rehmannia glutinosa Libosch. (prepared) | 熟地黄 (Shudihuang) | 16 | Catalpol; 5-hydroxymethylfurfural | HPLC–MS |
| 10 | Salvia miltiorrhiza Bge. | 丹参 (Danshen) | 16 | Danshensu; salvianolic acid B; tanshinone IIA | HPLC–MS |
|  | Total (ten herbs) |  | 139 |  |  |

All ten herbs were purchased from the pharmacy of the Third Affiliated Hospital of Guangzhou University of Chinese Medicine and authenticated by a licensed pharmacist. Marker compounds were those specified for the corresponding crude drugs in the Chinese Pharmacopoeia (2020 edition). The herbs are combined in the relative proportions shown (parts by weight, total 139 parts); a single lyophilised batch (batch No. 20250312) was prepared according to the hospital’s standardised protocol, the aqueous extract was freeze-dried, and the content of each marker compound was determined by HPLC–MS against the corresponding reference standard, with an acceptance limit of ±20% of the nominal content. The certificate of analysis for batch No. 20250312 is available from the corresponding author on request.
